# Australasian randomised trial to evaluate the role of maternal intramuscular dexamethasone versus betamethasone prior to preterm birth to increase survival free of childhood neurosensory disability (A*STEROID): study protocol

**DOI:** 10.1186/1471-2393-13-104

**Published:** 2013-05-03

**Authors:** Caroline A Crowther, Jane E Harding, Philippa F Middleton, Chad C Andersen, Pat Ashwood, Jeffrey S Robinson

**Affiliations:** 1 Australian Research Centre for Health of Women and Babies (ARCH), The Robinson Institute, Discipline of Obstetrics and Gynaecology, Women’s & Children’s Hospital, The University of Adelaide, 72 King William Road, North Adelaide, South Australia, 5006, Australia; 2 Liggins Institute, The University of Auckland, Auckland, New Zealand; 3 Department of Perinatal Medicine, Women’s and Children’s Hospital, Adelaide, Australia

**Keywords:** Antenatal corticosteroids, Dexamethasone, Betamethasone, Preterm birth, Randomised controlled trial, Neurosensory disability

## Abstract

**Background:**

Both dexamethasone and betamethasone, given to women at risk of preterm birth, substantially improve short-term neonatal health, increase the chance of the baby being discharged home alive, and reduce childhood neurosensory disability, remaining safe into adulthood. However, it is unclear which corticosteroid is of greater benefit to mother and child.

This study aims to determine whether giving dexamethasone to women at risk of preterm birth at less than 34 weeks’ gestation increases the chance of their children surviving free of neurosensory disability at two years’ corrected age, compared with betamethasone.

**Methods/Design:**

*Design* randomised, multicentre, placebo controlled trial.

*Inclusion criteria* women at risk of preterm birth at less than 34 weeks’ gestation with a singleton or twin pregnancy and no contraindications to the use of antenatal corticosteroids and who give informed consent.

*Trial entry & randomisation* at telephone randomisation eligible women will be randomly allocated to either the dexamethasone group or the betamethasone group, allocated a study number and corresponding treatment pack.

*Study groups* women in the dexamethasone group will be administered two syringes of 12 mg dexamethasone (dexamethasone sodium phosphate) and women in the betamethasone group will be administered two syringes of 11.4 mg betamethasone (Celestone Chronodose). Both study groups consist of intramuscular treatments 24 hours apart.

*Primary study outcome* death or any neurosensory disability measured in children at two years’ corrected age.

*Sample size* a sample size of 1449 children is required to detect either a decrease in death or any neurosensory disability from 27.0% to 20.1% with dexamethasone compared with betamethasone, or an increase from 27.0% to 34.5% (two-sided alpha 0.05, 80% power, 5% loss to follow up, design effect 1.2).

**Discussion:**

This study will provide high-level evidence of direct relevance for clinical practice. If one drug clearly results in significantly fewer deaths and fewer disabled children then it should be used consistently in women at risk of preterm birth and would be of great importance to women at risk of preterm birth, their children, health services and communities.

**Trial registration:**

Trial registration number: ACTRN12608000631303

## Background

Children born preterm have a higher than average risk of dying in the first weeks of life and survivors experience higher rates of neurosensory disability than children born at term [1,2]. There is clear evidence that antenatal corticosteroids, both dexamethasone and betamethasone, given to women at risk of preterm birth substantially improve short-term neonatal health, increase the chance of the baby being discharged home alive, and reduce childhood neurosensory disability, remaining safe into adulthood [3]. The type of antenatal corticosteroid to use in clinical practice remains unclear [3]. Dexamethasone and betamethasone are both recommended [4] and widely used [1].

Is dexamethasone better than betamethasone, or vice versa? Our meta-analysis, using the limited data available from randomised trials that have directly compared the two treatments shows that dexamethasone reduces the risk of the baby developing intraventricular haemorrhage (IVH) to a greater extent than betamethasone [5]. While reduced IVH is a very important outcome, achieving a reduction in long-term survival free of any disability is the most important goal [2]. Data regarding the effects on long-term health are sparse.

### Preterm birth and antenatal corticosteroids

One of the major advances in maternal fetal medicine over the last 35 years has been the evaluation and implementation into clinical practice of administration of antenatal corticosteroids to women at risk of preterm birth [3]. Corticosteroids remain the ‘gold standard’ antenatal intervention to reduce the risk of fetal and neonatal mortality related to preterm birth.

Meta-analysis of the 21 randomised trials (14 using betamethasone, six using dexamethasone, one unknown, using a wide range of different treatment regimens, in 3885 women and 4269 infants) included in the Cochrane Systematic Review “Antenatal corticosteroids for accelerating fetal lung maturation” [3] shows that, compared with placebo, both dexamethasone and betamethasone have large and clinically substantial beneficial effects compared with controls on the risk of fetal and neonatal death, respiratory distress syndrome, intraventricular haemorrhage (IVH) and long-term neurological sequelae [3]. Thirty years after antenatal betamethasone treatment, health outcomes are generally good, apart from indirect evidence of possible mild insulin resistance [6], but there have been no follow-up studies of similar duration for dexamethasone.

Clinical practice guidelines for the use of antenatal corticosteroids have been developed by many colleges and institutions [7,8], often based on the NIH Consensus Panel Guidelines that were first released in 1994 [9], and again in 2000 [4]. These guidelines state that “All pregnant women between 24 and 34 weeks gestation who are at risk of preterm delivery within 7 days should be considered candidates for antenatal treatment with a single course of corticosteroids” [4].

### Corticosteroid type and regimen in current clinical practice

There is considerable variation between countries as to whether dexamethasone or betamethasone is used in clinical practice. There are likely to be many reasons for these differences including availability, costs [10] (dexamethasone is cheaper than betamethasone so is widely used in low-income countries), impact of non-randomised, observational studies on efficacy and safety [11,12] and influence of opinion leaders [13]. In Australia and New Zealand both preparations are used, betamethasone most frequently [14].

The optimal treatment regimen to use for antenatal corticosteroids is uncertain. The current Cochrane review “Antenatal corticosteroids for accelerating fetal lung maturation” presents no evidence on an optimal dosing regimen [3]. An earlier version of this Cochrane review [15] mentioned “a policy of administering corticosteroids (24 mg betamethasone, or 24 mg dexamethasone)”, without specifying treatment regimens in detail. The NIH Consensus Statement, based on information available in the late 1990s, recommended “two doses of 12 mg of betamethasone given intramuscularly 24 hours apart or four doses of 6 mg of dexamethasone given intramuscularly 12 hours apart” [4]. Since 2000 there have been four further trials, some using different treatment regimens, and these are now included in the Roberts systematic review with the review authors highlighting that “further research is required to determine the optimal dose to use” [3].

Given this uncertainty it is not surprising to find a variety of different treatment regimens used in clinical care. For instance, to complete a course of antenatal corticosteroids, 48% of practitioners in Australia and New Zealand prescribe two injections at 12 hour intervals and 43% prescribe two injections at 24 hour intervals, with variation for both dexamethasone and betamethasone [14]. Practice is similarly varied in the UK [16]. To complete a course of 24 mg dexamethasone over 24 hours, the treatment regimen varies between institutions, and even within institutions, and includes 8 mg for 3 doses 8 hours apart [17], 12 mg for two doses 12 hours apart [17,18], or 12 mg for two doses 24 hours apart [18,19].

### Evidence from non-randomised cohort studies using dexamethasone or betamethasone

There have been several recent non-randomised, retrospective human studies comparing antenatal dexamethasone and betamethasone [11,12,20-22], and particularly their effects on IVH and periventricular leukomalacia; both major precursors for cerebral palsy. Results are conflicting, with some studies reporting no differences in the risk of IVH [12,21,22], periventricular leukomalacia [11,21,22], or mortality [12,21,22], but others report that dexamethasone is associated with an increase in periventricular leukomalacia [12] and neurosensory impairment [20]. The inherent risk of bias with retrospective study designs should limit the conclusions drawn for clinical practice, but this caution is not always mentioned [11,13,20-22]. Furthermore, it is possible that some effects of dexamethasone on periventricular leukomalacia may be attributable to the sulphite preservative used in some formulations rather than the drug itself, as shown in a mouse model [23].

### Appraising the evidence - the cochrane review

#### Evidence from randomised controlled trials using dexamethasone or betamethasone: effect on childhood health - reduction in neurosensory disability

Five randomised trials (908 children) with long-term follow-up are included in the Roberts Cochrane systematic review; [3] three comparing antenatal betamethasone with placebo [24-26], and two comparing dexamethasone with placebo [27,28].

Compared with placebo, both antenatal dexamethasone and betamethasone were associated with less developmental delay in childhood (Relative Risk (RR) 0.49, 95% Confidence Interval (CI) 0.24 to 1.00, 2 trials, 518 children), and a trend towards fewer children having cerebral palsy (RR 0.60, 95% CI 0.34 to 1.03, 5 trials, 904 children) [3]. Authors in the Roberts’ review emphasise that their review does not suggest evidence of a difference in risk of cerebral palsy between betamethasone and dexamethasone. Highlighting this research gap they state “Future studies are needed to determine the optimal drug” [3].

#### Evidence from randomised trials: direct comparison of dexamethasone with betamethasone

We have conducted a Cochrane systematic review to assess the use of different corticosteroids and regimens for accelerating fetal lung maturation for women at risk of preterm birth [5].

Five trials met the inclusion criteria [29-33]. No health outcomes of relevance for the mother were reported from any of the trials. For the infant, antenatal exposure to dexamethasone was associated with a significantly lower risk of IVH than exposure to betamethasone (3.5% versus 6.9%, RR 0.44 95% CI 0.21 to 0.92, 4 trials, 717 infants). No significant difference was seen for respiratory distress syndrome, severe intraventricular haemorrhage, periventricular leukomalacia or death. Information on childhood outcomes is limited to a single trial that reported one instance of neurosensory disability among only 12 infants admitted to neonatal intensive care unit and followed up at 18 months [33].

We conclude from this systematic review that high quality randomised controlled trials comparing dexamethasone and betamethasone are needed. Trials must be adequately powered to be able to detect important differences, not only in neonatal outcomes but also in survival and health into childhood.

#### Aims and objectives of this trial

The primary aim of this randomised controlled trial is to determine whether giving antenatal dexamethasone to women at risk of preterm birth at less than 34 weeks’ gestation increases the chance of their children surviving free of neurosensory disability at two years’ corrected age, compared with women given antenatal betamethasone.

#### Hypotheses

The primary hypothesis of the study is that, compared with betamethasone, antenatal dexamethasone given to women at risk of preterm birth (less than 34 weeks’ gestation) reduces the risk of death or any neurosensory disability, caused by impairments such as cerebral palsy, blindness, deafness or developmental delay, in their children at two years’ corrected age.

The secondary hypotheses are that compared with betamethasone, antenatal dexamethasone treatment has benefits *up to the time of primary hospital discharge*, relating to: *the baby,* of neonatal morbidity; and *the mother,* of maternal infections, and quality of life; and *at two years’ corrected age,* relating to individual components of the primary outcome and body size, respiratory morbidity, blood pressure, behaviour and general health of the child.

## Methods/Design

### Ethics statement

Ethics approval was granted by the Children’s Youth and Women’s Health Services Human Research Ethics Committee at the Women’s and Children’s Hospital (REC2074/7/14) and by the local institutional review boards for each centre.

### Study design

Randomised, multicentre, placebo controlled trial.

#### Inclusion criteria

Women are eligible for the trial if they are at risk of preterm birth at less than 34 weeks gestation, have a singleton or twin pregnancy, no contraindications to the use of antenatal corticosteroids and give informed consent.

#### Exclusion criteria

Women are not eligible if they have chorioamnionitis requiring urgent delivery, a higher order multiple pregnancy, have already received antenatal corticosteroids, have known fetal lung maturation, or are in the second stage of labour.

### Trial entry

All eligible women will be given the trial information sheet, counselled by a member of the research team and encouraged to discuss the study with her family before written informed signed consent is sought.

#### Study groups

Once all entry details are given and eligibility is confirmed, the woman is randomised by contacting the central telephone randomisation service at the University of Adelaide. Assignment to one of two study groups: either the dexamethasone group or the betamethasone group will be stratified for collaborating centre, gestational age (<28 weeks’, ≥28 weeks’ gestation), and number of fetuses (1 or 2). A Study Number will be allocated to the woman corresponding to a treatment pack, each of which looks identical and contains two opaque study-labelled syringes.

##### Dexamethasone study group

Women randomised to the dexamethasone study group will receive the contents of the two syringes intramuscularly 24 hours apart, each containing 12 mg dexamethasone (as dexamethasone sodium phosphate - a non-sulphite containing preparation).

##### Betamethasone study group

Women randomised to the betamethasone study group will receive the contents of the two syringes intramuscularly 24 hours apart, each containing 11.4 mg betamethasone (as Celestone Chronodose 11.4 mg).

##### Both study groups

At weekly intervals, if the woman has not yet given birth, and remains at continued risk of preterm birth, justifying the use of repeat antenatal corticosteroids [34-36], a ‘repeat treatment pack’ containing a single syringe from the same treatment group will be allocated using the telephone randomisation service.

Care during the antenatal period, labour and postnatal stay will be managed by the obstetric team caring for the woman. Care of the neonate will be the responsibility of the attending neonatologist.

### Follow up after birth until the time of primary discharge for both groups

The pregnancy and labour data will be abstracted from case notes by the masked research assistant at the collaborating hospitals. The postnatal and neonatal data will be collected similarly after discharge of the mother and baby from hospital.

### Longer term follow up

Parents enrolled in the trial will give consent for follow-up of their children from birth until two years’ corrected age at the time of the initial, prenatal recruitment. Mothers of all babies discharged home alive will be contacted by a member of the study team from the coordinating centre by mail soon after their baby is discharged and then again when their baby is six, 12 and 18 months corrected age. Mothers will be provided with a reply paid envelope and asked to confirm their current contact details by mail. At each of these times, a member of the study team at the coordinating centre will contact the parents by telephone if a response has not been received in the mail.

### Two years assessments

All surviving children will be formally assessed at two years of age, corrected for prematurity, by a developmental paediatrician and psychologist or other trained assessor who can administer the Bayley Scales, all of whom remain blinded to treatment group assignment. The parents will be contacted by the follow-up coordinator at the recruitment hospital to organise this appointment. However, if the family does not live close to this hospital, a member of the study team at the coordinating centre will telephone them to discuss a suitable alternative. Assessments will be made of health, neurodevelopment, behaviour, growth and blood pressure.

#### Motor function

The paediatric assessment will include a neurological examination to diagnose cerebral palsy (loss of motor function and abnormalities of muscle tone and power) and other disability outcomes according to previously reported criteria [37]. The severity of gross motor function in children will be classified using the Gross Motor Function Classification System (GMFCS) [38].

#### Psychological assessments

The psychological assessment will include the cognitive, motor and language scales of the Third Edition of the Bayley Scales of Infant Development (BSID-III) [39]. This is well-standardised with demonstrated validity and reliability. Psychological test scores will be recorded as a standardised normal score [derived from test score - mean/standard deviation (SD)]. Children with severe developmental delay who are unable to complete the psychological assessment will be given a standardised score of - 4 SD.

#### Behaviour

The child’s caregiver will be asked to complete the Child Behaviour Checklist [40].

##### General health, health resource utilisation, blood pressure, body size, and quality of life

A general history and physical examination will determine the presence of any significant chronic illness, and data regarding hospital readmissions will be confirmed, where necessary, from the admitting hospital or doctor. Children will be considered blind if visual acuity in both eyes is worse than 6/60. Children will be considered deaf if their hearing loss is sufficient to require hearing aid(s), or worse. Blood pressure will be measured and converted to Z-scores relative to American data for blood pressure for age, height and gender in childhood [41].

Questionnaires will be completed by the child’s caregiver about any respiratory morbidity, history of illness or injury and use of health services since primary hospitalisation.

The child’s height, weight, and head circumference will be measured in the standard way, and values for the relevant centile, percent of median, and standard deviation scores (Z scores) specific for age and gender will be computed from the British Growth Reference [42].

### Categorisation of neurosensory disability

Children will be considered to have a neurosensory impairment if they have cerebral palsy, blindness, deafness or any of the Bayley Scale scores more than 1 SD below the mean (<−1 SD). The neurosensory disabilities imposed by the various neurosensory impairments will be classified as severe, moderate or mild [37] (Table 1).

**Table 1:** **Neurosensory disability classifications**[37]


**Severe disability**	Any severe cerebral palsy (child non-ambulant and likely to remain so; GMFCS level 4 or 5), severe developmental delay (standardised score <−3 SD) or blindness.
**Moderate disability**	Moderate cerebral palsy (child non-ambulant at 2 years of age but who is likely to ambulate subsequently; GMFCS level 2 or 3), or deafness, or moderate developmental delay (standardised score from −3 SD to <−2 SD).
**Mild disability**	Mild cerebral palsy (child walking at 2 years of age with only minimal limitation of movement (GMFCS level 1), or suspect developmental delay (standardised score from −2 SD to <−1 SD).
**No neurosensory disability**	Children without any neurosensory impairment.

### Primary study endpoints

The primary study endpoint measured in the children at two years’ corrected age is the incidence of death or any neurosensory disability defined as stillbirths, deaths from live born infants before hospital discharge and deaths after hospital discharge; and any neurosensory disabilities that include the neurosensory impairments of cerebral palsy, blindness, deafness and any developmental delay defined as a standardised score more than 1 SD below the mean (<−1 SD).

### Secondary study endpoints

#### For the infant/child

•Death or major neurosensory disability (with major neurosensory disability defined as severe and moderate disability and includes blindness, deafness, cerebral palsy in a child non-ambulant by two years of age or a standardised score <−2 SD).

•Individual components and severity of the primary outcome (death, cerebral palsy, blindness, deafness, developmental delay).

•Neonatal outcomes measured prior to primary hospital discharge, including IVH, severe IVH (Grade 3 or 4), periventricular leukomalacia, retinopathy of prematurity needing treatment, patent ductus arteriosus needing treatment, respiratory distress syndrome, severity of any neonatal lung disease, chronic lung disease (need for oxygen at 36 weeks post-menstrual age or 28 days of life if born after 32 weeks gestation), use of mechanical ventilation, confirmed infection within the first 48 hours, infection after the first 48 hours, proven necrotising enterocolitis, body size at birth (weight, length and head circumference) and at discharge home after birth.

•Childhood outcomes assessed at two years’ corrected age including body size and corresponding Z scores, general health of the child (including use of health services since primary hospitalisation), childhood respiratory morbidity, blood pressure Z scores and proportions in hypertensive ranges and behaviour as assessed by the Child Behaviour Checklist [40].

#### For the mother

•Maternal perinatal infectious morbidity (defined as clinical chorioamnionitis requiring intrapartum antibiotics and/or use of postpartum antibiotics).

#### Sample size

With a predicted mortality rate of 6.05% up to two years and a predicted rate of neurosensory disability in survivors of 20.9%, we estimate the rate of our primary outcome of death or neurosensory disability at two years corrected age in an Australian/New Zealand population exposed antenatally to betamethasone to be 27.0% [3,37,43,44].

A trial of 1499 children, allowing 5% loss to follow up and with a design effect of 1.2 to allow for the clustering of babies within mothers, will have 80% power to detect a statistically significant difference at an alpha level of 0.05 (two-tailed) of either:

•a decrease in the combined outcome of death or neurosensory disability from 27.0% to 20.1% (Absolute Risk Difference (ARD) 6.9%; Relative Risk Difference (RRD) 25.6%) with dexamethasone compared with betamethasone, or;

•an increase from 27.0% to 34.5% (ARD 7.5%; RRD 27.8%).

#### Analysis and reporting of results

Data will be analysed by a statistician independent of the clinical investigators. Potential confounding variables will comprise sociodemographic variables, such as ethnicity, language spoken at home, family structure, mother’s marital status, social class, and mother’s and father’s education, as well as gender. Comparisons will be made between treatment groups for the primary and secondary endpoints using an intention to treat approach. Analyses will make adjustments for the stratification variables and for important baseline predictors including gestational age at trial entry, and reasons for risk of preterm birth. Unadjusted analyses will also be performed. Log binomial regression and linear regression will be used to examine dichotomous and continuous outcomes respectively, with results presented as relative risks or differences in means along with 95% confidence intervals. Adjustment will be made for clustering due to multiple births for infant outcomes using generalised estimating equations. *P*-values < 0.05 will be considered statistically significant.

## Discussion

Administration of antenatal corticosteroids, both dexamethasone and betamethasone, given to women at risk of preterm birth, leads to substantial benefits for babies born preterm. Although this cost-effective treatment is widely used in practice throughout the world, there is uncertainty as to which drug treatment is of greatest benefit. Clearly, large, high quality randomised controlled trials are a priority to provide high-level evidence for clinical practice. If one drug clearly results in significantly fewer deaths and fewer disabled children then it should be used consistently in women at risk of preterm birth and would be of great importance to women at risk of preterm birth, their children, health services and communities.

## Abbreviations

ARD: Absolute risk difference; CI: Confidence interval; GMFCS: Gross motor function classification system; IVH: Intraventricular haemorrhage; RR: Relative risk; RRD: Relative risk difference; SD: Standard deviation

## Competing interests

The authors declare they have no competing interests.

## Authors’ contributions

CAC, JEH, PFM, CCA, PA and JSR are all members of the ASTEROID Study Group. The primary investigator of the ASTEROID Study (CAC) wrote the first draft of the ASTEROID protocol and prepared the initial draft. CAC, JEH and PFM conceived the study and participated in the project design. All authors were involved in the development of the design of the study, the protocol development, have commented on all drafts of the protocol, and have read and approved the final draft of the protocol. All authors read and approved the final manuscript.

## Pre-publication history

The pre-publication history for this paper can be accessed here:

http://www.biomedcentral.com/1471-2393/13/104/prepub
